# Resistance to anoikis in transcoelomic shedding: the role of glycolytic enzymes

**DOI:** 10.1515/pp-2019-0003

**Published:** 2019-03-12

**Authors:** Robert B. Wilson, Wiebke Solass, Rami Archid, Frank-Jürgen Weinreich, Alfred Königsrainer, Marc A. Reymond

**Affiliations:** Department of Surgery, University of South New Wales, Sydney, Australia; Institute of Pathology, University of Tübingen, Tübingen, Germany; Department of General and Transplant Surgery, University of Tübingen, Tübingen, Germany; Department of Upper Gastrointestinal Surgery, Liverpool Hospital, Elizabeth St, Liverpool, Sydney, New South Wales, Australia

**Keywords:** anoikis, autophagia, glycolysis, inflammation, peritoneal metastasis, phosphoglycerate kinase 1

## Abstract

Detachment of cells from the extracellular matrix into the peritoneal cavity initiates a cascade of metabolic alterations, leading usually to cell death by apoptosis, so-called *anoikis*. Glycolytic enzymes enable the switch from oxidative phosphorylation to aerobic glycolysis and allow resistance to anoikis of shed tumour cells. These enzymes also have moonlighting activities as protein kinases and transcription factors. Phosphoglycerate kinase (PGK) and pyruvate kinase are the only glycolytic enzymes generating ATP in the hexokinase pathway. Hypoxia, EGFR activation, expression of K-Ras G12V and B-Raf V600E induce mitochondrial translocation of phosphoglycerate kinase 1 (PGK1). Mitochondrial PGK1 acts as a protein kinase to phosphorylate pyruvate dehydrogenase kinase 1 (PDHK1), reducing mitochondrial pyruvate utilization, suppressing reactive oxygen species production, increasing lactate production and promoting tumourigenesis. PGK1 also plays a role as a transcription factor once transported into the nucleus. Resistance to anoikis is also facilitated by metabolic support provided by cancer-associated fibroblasts (CAFs). Our series of experiments in-vitro and in the animal model showed that PGK1 knock-out or inhibition is effective in controlling development and growth of peritoneal metastasis (PM) of gastric origin, establishing a causal role of PGK1 in this development. PGK1 also increases CXCR4 and CXCL12 expression, which is associated with a metastatic phenotype and plays a role in the metastatic homing of malignant cells. Thus, PGK1, its modulators and target genes may be exploited as therapeutic targets for preventing development of PM and for enhancing cytotoxic effects of conventional systemic chemotherapy.

## Introduction

Peritoneal metastasis (PM) results from shedding of tumour cells from the primary malignancy, transport through the peritoneal cavity, adhesion to the peritoneum, invasion, proliferation, and angiogenesis [1]. Presence of malignant cells in the peritoneal cavity is a frequent observation and has been reported in 22% patients with colorectal cancer [2], 10% with pancreas cancer [3] and 6% patients with gastric cancer undergoing perceived curative resection [4]. Positive cytology has usually been associated with a dismal prognosis [5]. The rate of intraperitoneal recurrence and peritoneal metastasis after curative surgery is high, for example 80% in ovarian cancer and 50% in gastric cancer [6].

Progression of PM is enhanced by [1, 7, 8]:

1. Direct **cell-cell contact**, for example adhesion of RHAMM/CD44 positive cancer cells onto mesothelial hyaluronic acid (HA) and interactions between mesothelial cell surface fibronectin and cancer cell-derived α5β1 integrins, adhesion of IL-1β expressing cancer cells onto mesothelial β1 integrins, formation of spontaneous cancer spheroids, and cancer cells squeezing past mesothelial gap junctions to access the underlying basement membrane (BM) and extracellular matrix (ECM).
2. **Paracrine activity** of soluble factors released by peritoneal mesothelial cells (PMC): cytokines (IL-1, IL-6, IL-15), chemokines (CXCL8/IL-8, CCL2/MCP-1, RANTES, CXCL1/GRO-1, CXCL12/SDF-1), growth factors (TGF-β1, PDGF, FGF, EGF, VEGF), ECM elements (collagens I, III, IV, fibronectin, elastin, vitronectin), and adhesion molecules (ICAM-1, VCAM-1, E-cadherin).
3. **Cancer cell contact with the ECM:** IL-1β expression and exposure of submesothelial collagen, blood vessels, and sequestered VEGF/TGF-β1 enhance cancer cell adhesion and invasion, particularly at peritoneal stomata/omental milky spots.
4. **Ascites** nourishing detached cells, providing transport for dissemination of cancer spheroids, preparing the peritoneum for cancer cell adhesion by spreading soluble growth factors/adhesion molecules/chemokines, and allowing a permissive immunoenvironment (TGF-β1, TAMS and IL-17).

## Anoikis

The presence of tumour cells within the peritoneal cavity does not always lead to metastatic disease. Some patients with positive cytology survive for years without developing intraperitoneal tumour recurrence [3]. Several mechanisms may explain this phenomenon, such as the inability of most detached cancer cells to survive outside the ECM of the primary tumour (*anoikis)* [9], autophagy and tumour dormancy, immunological defense mechanisms within the peritoneal cavity [10] and the barrier offered by the peritoneal mesothelium and its protective glycocalyx [11].

**Figure 1:**
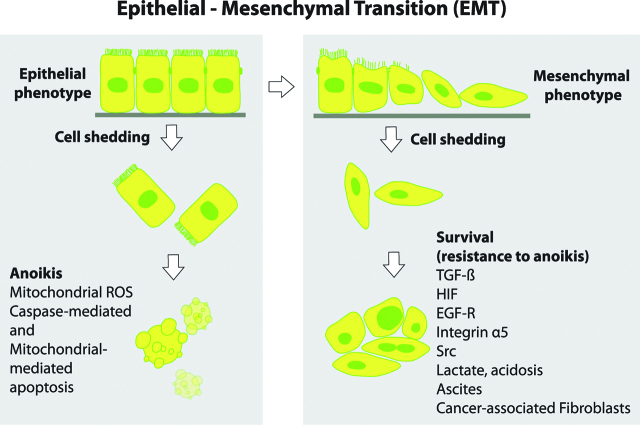
EMT and resistance to anoikis. Normal epithelial and premalignant cells undergo programmed cell death via *anoikis* upon detachment from the extracellular matrix/ tumor microenviroment (TME). Cancer cells develop resistance to anoikis and are able to survive in the peritoneal cavity despite shedding from the ECM/basement membrane. Key drivers of resistance to anoikis include TGF-β, Hypoxia-inducible factor (HIF), Epidermal-growth factor Receptor (EGF-R), Src, low pH, lactate, integrin α, heat shock protein (HSP), cytokines and the presence of Cancer-associated fibroblasts (CAFs) and ascites in the peritoneal cavity (Adapted from [15]).

Recently it was shown that detached cancer cells spontaneously form tumour spheroids with floating peritoneal fibroblasts/Cancer Associated Fibroblasts (CAFs) in the peritoneal cavity, which enhances their survival, dissemination, migration ability and invasion potential. Peritoneal cancer heterospheroids are made up of a backbone of EGF secreting CAFS surrounded by epithelial cancer cells expressing EGF-R. The cancer cells respond to the secreted EGF or TGF-β1 by expressing integrin α5, which further activates the CAFs. Disaggregation of tumour heterospheroids to allow adhesion to the peritoneum requires proteolysis of fibronectin and vitronectin by activated matrix metalloprotease 2 (MMP-2). This compares to detached or sparse cancer cells that do not contact peritoneal fibroblasts – these are more likely to not survive anoikis and thus undergo apoptosis [12]. Damaged or senescent peritoneal mesothelium is also more receptive to cancer cell adhesion and implantation than young or undamaged mesothelium. This is observed in ovarian, gastric and colorectal peritoneal metastasis [1, 13, 14] (Figure 1).

## Resistance to anoikis

In normal tissues, epithelial cells require attachment to the ECM for survival. The process of epithelial-mesenchymal transition (EMT) takes centre stage as tumour cells physically detach from their primary tumour and develop mesenchymal properties [16] (Figure 1). Detachment of cells from the ECM initiates a cascade of metabolic alterations, including defective glucose uptake, diminished pentose phosphate pathway (PPP) flux, reduced cellular ATP levels, and an increase in mitochondrial-generated reactive oxygen species (ROS) [17]. This usually leads to anoikis and cellular death by apoptosis. In contrast, to be able to survive outside the ECM of the primary tumour, detached cancer cells need to acquire resistance to programmed cell death. This ability to evade apoptosis is known as resistance to anoikis [18] and is characterized by diverse pro-survival cellular and metabolic changes.

### Resistance to anoikis: cellular changes

Loss of cellular attachment to the ECM normally results in activation of cell surface anoikis-initiating molecules such as integrin and death receptors. Anoikis induces apoptosis by either the caspase-8-mediated extrinsic apoptosis signalling pathway or the mitochondrial-mediated intrinsic apoptosis signalling pathway [19]. Resistance to anoikis is fundamental to the ability of cells to avoid apoptosis, acquire migratory ability and intravasate.

**Figure 2:**
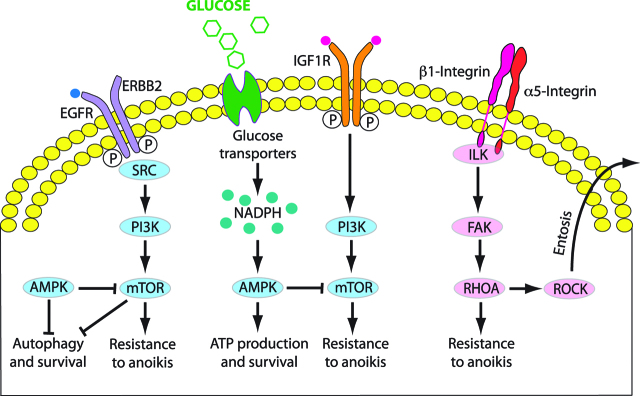
Mechanisms of resistance to anoikis. Survival strategies used by extracellular matrix (ECM)-detached metastatic cancer cells and corresponding signaling pathways (Adapted from [20]).

Cellular mechanisms of survival of detached metastatic cells involve IGF1-R, ERBB, EGF-R and integrin receptors (reviewed in [20]). During hypoxia, the actin cytoskeleton is regulated and modified via Rho GTPase signalling. Rho is a family of small guanosine triphosphatases (Rho GTPases) associated with actin cytoskeleton in several cell types and tissues, and induce actin rearrangement and actin stress fibre assembly under hypoxic conditions (reviewed in [21]). This enables dynamic formation of finger-like filopodia and microvilli; and sheet-like lamellipodia and ruffles. These membrane protrusions are able to attach to and degrade the ECM, and are important in both proteolytic migration and amoeboid migration of cells [22, 23]. Pseudopodial protrusions are Rho/Rho associated protein kinase (ROCK)-dependent and promoted by phosphorylation of caveolin 1 and activated Src, a family of proto-oncogenic tyrosine kinases which enables cancer cell polarization, cell motility and invasion [24]. Activation of Src by EGF leads to phosphorylation of the scaffold protein Tks5, also known as SH3PXD2a/FISH. Tks5 is indispensable for matrix proteolysis by the invasive pseudopodia of transformed PMC on the invading edge of cancer tissue in the peritoneum [23] (Figure 2).

The phosphatidylinositol 3-kinase (PI3K)/AKT/mammalian target of rapamycin (mTOR) signalling pathway is hyperactivated or altered in many cancer types and increases expression of downstream targets contributing to anoikis resistance (reviewed in [25]). Upon detachment of cancer cells from the BM/ECM, NOX4 is upregulated by TGF-β1 and angiotensin II and induction of EMT via the TGF-β1-NOX4 axis occurs via p38 MAPK signalling [26, 27]. Mitochondrial complex III generation of ROS is required for K-Ras induced anchorage-independent growth via the ERK/MAPK signalling pathway and activation of tyrosine kinase Src [28].

Overexpression of MUC-1, frequently observed in PM, also contributes to resistance to anoikis by enabling shed cells to survive oxidative stress [27]. In cancer, mucins often take on an oncogenic role and promote a number of tumourigenic effects, including pro-survival, migratory, and invasive behaviours [29]. MUC1 physically interacts with HIF-1α to stabilize the protein and then binds to the promoter of multiple glycolytic genes to enhance their expression in a hypoxia-dependent manner. MUC1 mediated-target genes enhance glucose uptake and metabolism, thus facilitating cancer cell survival and growth by glycolysis related metabolism [30].

### Resistance to anoikis: metabolic changes

**Figure 3:**
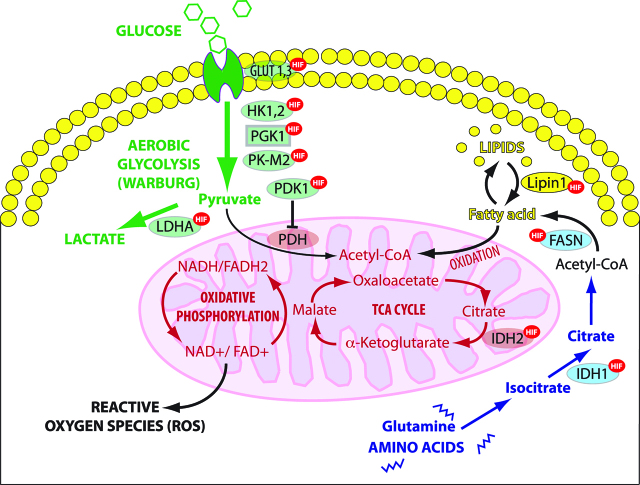
Regulation of glycolysis and TCA cycle by HIF. HIF-1 enhances aerobic glycolysis in the cytoplasm by which glucose is metabolized to lactate rather than converted to acetyl-CoA to undergo oxidative phosphorylation (OXPHOS) in mitochondria. HIF-1 stimulates the utilization of glutamine and fatty acids as alternative substrates for mitochondrial energy production. OXPHOS: oxidative phosphorylation, TCA cycle: tricarboxylic acid or Krebs cycle; ROS: reactive oxygen species, LDHA: lactate dehydrogenase A, IDH: Isocitrate dehydrogenase, FAD: Flavin adenine dinucleotide, FASN: fatty acid synthase, NAD: Nicotinamide adenine dinucleotide, PDH: pyruvate dehydrogenase; PDK1: pyruvate dehydrogenase kinase isozyme 1, PK-M2: pyruvate kinase muscle isozyme 2; PGK1: Phosphoglycerate kinase 1 HK1,2: Hexokinase-1,2 (Adapted from [39]).

Cancer cells can alter their metabolism according to the tumour microenvironment (TME), available nutrients and their level of oxidative stress. They can generate energy through both aerobic glycolysis and mitochondrial oxidative phosphorylation at the same time. The Warburg effect, named after its discoverer Otto Warburg [31], refers to the observation that cancer cells preferentially use aerobic glycolysis in the cytoplasm as a source of energy rather than the more efficient mitochondrial pathway of oxidative phosphorylation (Figure 3). Although aerobic glycolysis is 90% less efficient as mitochondrial oxidative phosphorylation in producing ATP, it is 10–100 times more rapid. Therefore, by greatly upregulating the glucose transporters, glycolytic enzymes and lactate production, a comparable amount of ATP may be generated to enable cell survival [32].

Under physiological conditions, oxygen and glucose content of the peritoneal cavity is low, leading to decreased ATP and pyruvate production in ‘homeless’ cells. In the presence of peritoneal metastasis, the intraperitoneal glucose level is diminished, whereas lactate concentration increases [33]. Thus, cancer cells shed into the peritoneal cavity must switch from aerobic mitochondrial respiration to cytoplasmic glycolysis as an alternative ATP source in order to resist anoikis. For example, in the presence of oxygen, 91 % of cancer cell ATP is generated by mitochondrial oxidative phosphorylation, but under hypoxic conditions this is only 36%.

### Oncogenic pathways involved in the glycolytic switch

Main regulators of the *glycolytic switch* observed in cancer cells involve HIF-1α, c-Myc, Akt and mTOR [34]. Other regulators include K-Ras, tumour suppressor p53, energy sensor adenosine monophosphate activated protein kinase (AMPK) and SIRT.

– HIF-1α

HIF is a transcription factor involved in catabolic cellular processes under hypoxic conditions. The major role of HIF-1α in pathogenesis of chronic inflammation and peritoneal metastasis has been reviewed recently in this journal [32]. HIF-1 regulates glucose, lipid, and glutamine metabolic flux in cancer cells. HIF-1α is a major actor in the regulation of glycolysis, in particular in activating both anaerobic and aerobic glycolysis and lactate production. Upregulation of HIF-1-mediated hexokinase II (HKII) results in a high glycolytic rate in hypoxic solid tumours [35]. HIF-1α potentiates the transcription of glucose transporters (GLUT) and glycolytic enzymes including, HKII, pyruvate dehydrogenase kinase (PDK), and pyruvate kinase muscle isozyme 2 (PKM2) [36, 37, 38]. Thus, glucose is metabolized to lactate in the cytoplasm rather than converted to acetyl-CoA for oxidative phosphorylation (OXPHOS) in mitochondria. As a result, cancer cells are protected against excessive ROS stress generated from electron transport chain (ETC) when the oxygen supply is limited. To sustain the tricarboxylic acid (TCA) cycle and meet the biosynthetic demands, HIF-1 stimulates the utilization of glutamine which, by activating a reductive carboxylation pathway, enters the TCA (Krebs) cycle and induces fatty acid synthesis for rapid cell growth and division [39] (Figure 3).

– C-Myc

In cancer cells, metabolism is interconnected with the cell cycle, nuclear transcription and EMT, and cell migration. Glycolytic enzymes such as PKM2, 6-phosphofructo-2-kinase/fructose-2,6-biphosphatase (PFKFB3) and GAPDH can translocate to the nucleus and regulate cell cycle or oncogene expression, including c-Myc. c-Myc upregulates glucose transport (GLUT-1), transcription of HKII, phosphofructokinase (PFK) and lactate dehydrogenase A (LDHA). C-Myc also promotes a high PKM2/PKM1 ratio (reviewed in [40]). Inactivation of pyruvate kinase M2 (PKM2) promotes transcription in G1 phase, and glutamine metabolism supports DNA replication in S phase and lipid synthesis in G2 phase.

C-Myc also upregulates the monocarboxylate transporter (MCT) family. MCT determine uptake and release of cellular catabolites such as monocarboxylates: lactate, pyruvate, branched chain amino acids and ketone bodies. MCT4 is involved in the release of monocarboxylates from cells, whereas MCT1 is predominantly involved in the uptake of these catabolites. MCT4 is regulated by HIF-1α and NF-κB and is highly expressed in CAFs, allowing shuttling of lactate between CAFs and cancer cells [41].

– mTOR

Cancer cells utilize autophagy or mitophagy to recycle damaged or unwanted organelles and macromolecules and in so doing, generate energy and recover precursor building blocks needed for growth. mTOR-C1 maintains nutrient homeostasis through lysosomal biogenesis and autophagic processes [42]. mTOR is a serine-threonine kinase downstream of Akt (protein kinase B), which upregulates PKM2, an isoenzyme of the glycolytic enzyme pyruvate kinase, and GLUT3, a glucose membrane transporter. mTOR also activates HIF-1α, NF-κB, and c-Myc. The transmembrane tyrosine kinases (TKR) activate PI3K, which in turn recruits and activates Akt [36].

– K-Ras

In mammalian cells, the PPP and one-carbon metabolism are major sources of NADPH production [43]. NADPH is a reducing agent which provides the cell with ROS scavengers for control of redox stress, particularly in detached cancer cells, thus contributing to resistance to anoikis. NADPH is also necessary for biomass formation in rapidly dividing cells. The K-ras protein is associated with mitochondria, and induces a rapid suppression of respiratory chain complex-I and a decrease in mitochondrial transmembrane potential by affecting the cyclosporin-sensitive permeability transition pore. Mutated K-Ras leads to upregulation of GLUT1, cell survival in low glucose conditions, and direction of glycolytic metabolites down the non-oxidative PPP [34, 44].

– p53

The tumour suppressor p53 regulates downstream targets that determine cell fate. Canonical p53 functions include induction of apoptosis, growth arrest, and senescence. Non-canonical p53 functions include promotion or inhibition of autophagy and regulation of metabolism. Specifically, p53 decreases glucose transport (by downregulating GLUT1, GLUT3 and GLUT4) and also directly inhibits Glucose-6-phosphate-dehydrogenase (G6PD), which is the first and rate-limiting step of PPP. P53 mutations, EGF and PDGF *increase* glycolysis by increasing G6PD activity and glucose flux down the PPP [34, 43, 45]. P53 also inhibits PFK-1 activity via transcriptional upregulation of TIGAR (TP53-induced glycolysis and apoptosis regulator).

## Acidic extra cellular microenvironment

The regulation of cellular glycolysis and oxidative phosphorylation is pH-dependent. Normal cells have an acidic intracellular pH (pHi) and basic extracellular pH (pHe). An alkaline pHi promotes glycolysis, increases the activity of key glycolytic enzymes such as phosphofructokinase (PFK-1) and inhibits gluconeogenesis. The pHi/pHe ratio is reversed in cancer cells, where a sustained basic intracellular pH increases the activity of PFK-1 more than 100-fold. Again, the metabolic switch from mitochondrial respiration to glycolysis under hypoxia leads to the upregulation of HIF, TGF-β1, LDHA and the conversion of pyruvate to lactate instead of Acetyl-CoA [46]. For example, in ovarian cancer patients, peritoneal fluid glucose level was 3.67±0.21 mmol/l (vs. 4.76±0.14 mmol/l in benign ovarian disease), the glucose level was even lower in stage FIGO III and IV. In contrast, peritoneal fluid lactate was increased in ovarian cancer (1.68±0.08 mmol/l vs. 0.73±0.09 mmol/l in benign disease) [33].

Generation of an acidic extracellular microenvironment is a fundamental requirement for cancer cells to survive outside the ECM and undergo EMT. EMT is characterized by loss of E-cadherin, HIF/TGF-β1 metabolomic reprogramming, the Warburg effect, AGE-RAGE interactions, increased cytosolic glycolysis, inhibition of pyruvate decarboxylation by PDHK1 in the mitochondria, production of lactate from pyruvate by LDHA, and lactate shuttling by stromal MCT4. Failure of conversion of pyruvate to Acetyl Co-A enhances the production of ribose, glycerol and lactate, as this maintains glucose flux down the hexokinase pathway and the PPP. The conversion of pyruvate to lactate in the cytosol also generates NAD^+^, which is required for the continued conversion of glyceraldehyde-3-P to 1,3-biphosphoglycerate by GAPDH, particularly when there is not enough oxygen for NADH re-oxidation in mitochondria [47]. An alkaline pHi and acidic pHe is maintained by membrane-bound proton transporters (MBPT), mainly the Na^+^/H^+^ exchangers but also carbonic anhydrases (CAs, mainly CA IX and XII), vacuolar H^+^ ATPases, the H^+^/Cl^−^ symporter, the monocarboxylate transporters (MCT1), also known as the lactate-proton symporter, the Na^+^ dependent Cl^-^/HCO3^-^ exchanger or bicarbonate transporter and the ATP synthase [46].

Extracellular lactic acidosis promotes TGF-β1 release, genome instability, lamellipodia formation and β1-integrin activation; and cysteine protease, MMP-9 and glycosidase breakdown of the ECM. This enables tumour cell intravasation, angiogenesis, chemotherapy drug resistance, and immune evasion [48]. The importance of the pHi/pHe gradient in cancer cells is emphasized by the blockade of LDHA resulting in decreased tumourogenesis. The transcription of LDHA is regulated by lactate, HIF1, C-Myc, FOXM1, KLF4, cAMP, estrogen, ErbB2, Heat shock factor 1 (HSF1), FGFR1 and SIRT2 [49].

## Reverse Warburg effects

**Figure 4:**
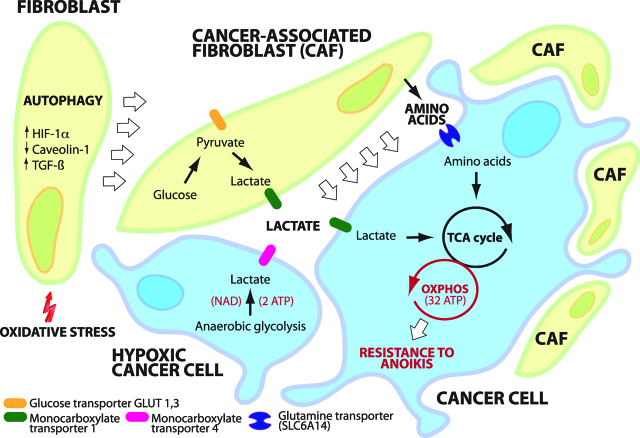
Reverse Warburg effect. Cancer associated fibroblasts (CAFs) form spheroids with cancer cells shed into the peritoneal cavity, forming “Metastatic Units” (MU). Autophagy of fibroblasts provides cancer cells with nutrients and macromolecular building blocks. CAFs support energy metabolism of cancer cells by supplying them with lactate and amino acids. Aerobic glycolysis in cancer cells is more efficient (mitochondrial oxidation: 32 molecules ATP/molecule Glucose; aerobic glycolysis: 4 molecules ATP/molecule Glucose) than anaerobic glycolysis (2 molecules ATP/ molecule Glucose). MU facilitate resistance of cancer cells to anoikis (12).

The *Reverse Warburg effect* describes the synergism between cancer cells and the TME, in particular stromal fibroblasts and CAFs. For detached cancer cells, one way of resisting anoikis is to recruit and transform PMCs and other host cells (endothelial cells, pericytes, resident fibroblasts, bone marrow stem cells, adipocytes) into glycolytic fibroblasts (CAFs). This recruitment is related to cancer cell k-ras expression, hydrogen peroxide excretion, loss of caveolin-1 and induction of CAF oxidative stress, HIF-1α, NF-κB and autophagy. Platelet derived TGF-β1 and PDGF in the TME, together with cancer cell conditioned media contribute to glycolytic stromal fibroblast (CAF) transformation. CAFs derived from transformed PMCs form the majority of cells at the invasive edge of the cancer tissue in PM, and express Tks5, a substrate of Src kinase. They penetrate the peritoneum by formation of “invadopodia” and MMP proteolysis of the ECM in the direction of invasion *ahead of cancer cells* [50]. Much of the ^18^F-FDG uptake in PET scanning of solid tumours can be attributed to the activity of transformed glycolytic fibroblasts and the reverse Warburg effect (Figure 4).

Nutrient deprivation in the TME promotes CAF autophagy and mitophagy via complex interactions between HIF1, TGF-β1, CTGF, mTORC1 and Phosphoglycerate kinase (PGK). Mitophagy reduces the number of dysfunctional CAF mitochondria and thence the generation of mitochondrial ROS, but also provides nutrients by autophagosomal degradation (Figure 4). These nutrients include lactate, 3-hydroxy-butyrate (a ketone body), and glutamine being extruded into the TME where they can fuel oxidative mitochondrial metabolism in adjacent cancer cells [51]. This paracrine stromal-epithelial cancer metabolic coupling is part of the ‘reverse Warburg effect’, which is dependent on upregulation of glucose transport (GLUT-1) and the glycolytic enzymes HK2, PKM2, LDH, enolase, and fructose-bisphosphate aldolase A in CAFs, together with the release of exosomal IL-6 and hydrogen peroxide from cancer cells. Once implanted, cancer cells re-establish oxygen supply via VEGF-induced neoangiogenesis and transformed stromal cells to produce lactate. CAF glycolysis occurs then in the *presence* of oxygen, referred to as aerobic glycolysis. Tyrosine phosphorylation of PKM2 by Src is important for aerobic glycolysis in caveolin-1 negative CAFs [52].

Glucose uptake is enhanced in CAFs compared to non-activated fibroblasts (NAFs), even when co-cultured with cancer cells [41, 53]. Metabolic coupling and shuttling of lactate and glutamine from CAFs to cancer cells allows for glucose in cancer cells to be directed into the PPP. This promotes cellular proliferation, biomass formation and reduction of oxidative stress in cancer cells. The thiamine pyrophosphate/transketolase dependent oxidative branch of the PPP converts one molecule of glucose-6-phosphate to ribulose-5-phosphate and produces 2 molecules of NADPH. NADPH is used for glutathione regeneration, ROS scavenging, fatty acid and cholesterol synthesis, as well as ribose production for nucleic acid and nucleotide synthesis in rapidly dividing cells. In the non-oxidative branch of the PPP, R-5-P in converted to F-6-P and G-3-P, which can be re-directed to glycolysis to produce pyruvate (so-called MODE IV of the PPP) [43].

Lactate generated by CAF glycolysis is excreted by MCT-4, re-absorbed via cancer cell MCT-1 and converted back to pyruvate by cancer cell LDHA to then be oxidized in the TCA cycle. Inhibition of MCT-1 under low glucose conditions leads to cancer cell apoptosis. This is analogous to lactate shuttling between glycolytic astrocytes and neurons, which spares neuronal glucose to be utilized in the neuronal PPP to produce NADPH, regenerate glutathione and minimize synaptic oxidative stress [47, 54, 55].

Similar to lactate, glutamine is produced by glycolytic CAFs and transported to be used by cancer cells in the TCA as a way to maintain mitochondrial activity, minimize intracellular ROS production and spare glucose. In co-culture with CAFs, the cancer cells show:

1. reduced glutamine synthesis as determined by glutamate amine ammonia ligase (GLUL),
2. increased glutamine catabolism as determined by glutaminase (GLS) and glutamate dehydroxylase 1 (GLUD1),
3. increased expression of a glutamine uptake transporter (SLC6A14) [56].

## Metabolic drivers of intraperitoneal tumour implantation

There are preferred locations for intraperitoneal tumour adhesion and growth, as exemplified by the greater omentum. Omental milky spots provide an hypoxic niche mediated by HIF-1α for the implantation, nutrition and proliferation of detached cancer stem cells. The omentum consists mostly of adipocytes- these are able to induce chemotaxis by adipokine secretion and to release fatty acids, thus providing energy substrates for cancer cells shed into the peritoneal cavity in the absence of glucose [57] Stored triglycerides in omental adipocytes undergo lipolysis to provide free fatty acids and glycerol. Beta oxidation of these FFA (FAO) can be used by cancer cells to feed NADH and FADH2 into the mitochondrial electron-transport chain to produce acetyl-CoA for the TCA cycle. Transport of fatty acids across the mitochondrial membrane is reliant on carnitine palmitoyltransferase 1 isoforms A and C (CPT1A and C), which are overexpressed in many human tumours [22, 58]. This is particularly important when the TME is hypoxic or deprived of glucose’ such as in the peritoneal cavity. Production of triglycerides is part of the glycolytic pathway and stems from fructose 1,6 BP conversion to Dihydroxyacetone phosphate (DHAP) and glyceraldehyde-3-phosphate. This occurs two steps before 1,3 biphosphoglycerate conversion to 3-phosphoglycerate by PGK.

## Non-metabolic functions of glycolytic enzymes: inflammation, immune response and autophagy

Recent research has led to the identification of several non-metabolic functions of glycolytic enzymes [59, 60] and in particular their role in inflammation and immunity. Effective immune-mediated inflammatory responses in the TME require upregulation of glycolytic enzymes in order to satisfy the metabolic requirements of the immune effector cells.

### Inflammation

In PM patients, nutritional parameters, including albumin and total protein serum levels. deteriorate until death. Body resting metabolism increases in the last months of life in parallel to increased tumour metabolism and systemic inflammation. Progressive anorexia impairs uptake of nutrients and constitutive body components are increasingly used as energy sources, leading to cachexia and ultimately death by lack of metabolic resources [61]. The Warburg and reverse Warburg effects may, in part, explain the cachexia-anorexia syndrome seen in patients with advanced PM. Weight loss is probably exacerbated by peritoneal carbonyl stress or continued IL-1 release, which cause anorexia and food avoidance. Inflammatory cytokines IL-1β and TNF‐α act synergistically with TGF‐β 1 in upregulating VEGF and IL‐6 production in PM. Cancer cachexia and related sarcopenia may also be related to TGF-β1 stimulation of myostatin, activin A and activin B, which results in profound muscle wasting [62, 63].

Several enzymes of the glycolytic pathway contribute to the inflammatory response (reviewed in [64]). For example, elevated glycolytic activity has been documented in T-cells during the inflammatory response. Generation of enough ATP and of metabolic intermediates is needed for anabolic processes in cancer tissue – a wound that does not heal [65].

### Autophagy

Glycolytic enzymes, including Phosphoglycerate kinase 1 (PGK1), are also involved in autophagy, a recycling process of damaged organelles to generate energy and reuse macromolecules for normal growth. PGK1 kinase activity is involved in the initiation of autophagy. This occurs when tumours start outgrowing existing vasculature or when cells detach into the coelom, resulting in glutamine deprivation and hypoxia within tumour cells. In this initial phase, ARD1 acetyltransferase acetylates PGK1 at Lys388, which in turn phosphorylates Beclin1 at Ser30, leading to a conformational change and activation of class III phosphatidylinositol (PI) 3-kinase (VPS34). The resulting production of phosphatidylinositol 3-phosphate (PI(3)P) facilitates the initiation of tumour autophagy and indirectly tumour development [66].

### Immunity

LDHA and tumour derived extracellular lactate promote cancer cell evasion of peritoneal T cell immunosurveillance by:

1. strongly inhibiting the proliferation and activity of human cytotoxic T lymphocytes (CTLs),
2. inducing release of TGF-β1, HIF-1α and immune checkpoint inhibitors (PD-L1),
3. suppression of IL-17 producing CD8^+^ T (Tc17) cells,
4. accumulation of myeloid-derived suppressor cells (MDSCs) while suppressing the function of natural killer (NK) cells and T lymphocytes,
5. M2 macrophage polarization [67].

In pre-clinical models, proton pump inhibitors – which inhibit extracellular acidification of the TME – have been shown to limit tumour expansion [68] and to restore functionality of tumour-infiltrating lymphocytes (TILs) and thus enhance therapeutic efficacy of adoptive immunotherapy [69]. Similarly, increasing the alkalinity of the TME by bicarbonate administration has improved therapeutic response to anti-PD-1 antibodies. Thus, counteracting tumour acidity of the TME may be used as an adjunct to enhance tumour response to immunotherapy [70].

## Role of glycolytic enzymes as nuclear transcription factors

**Figure 5:**
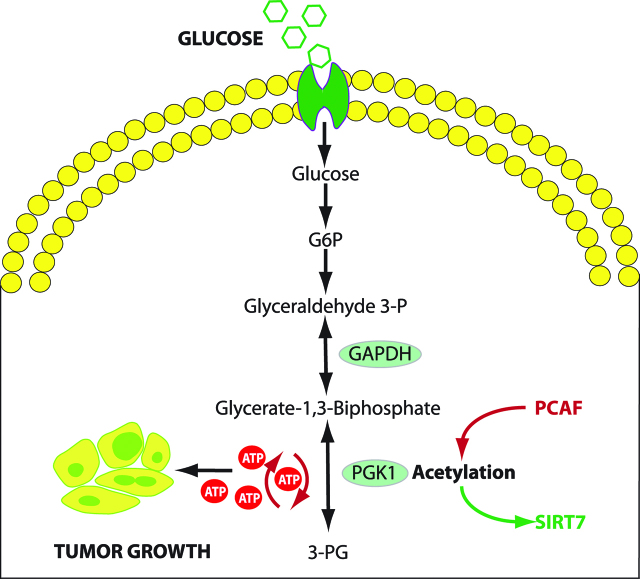
PGK1 activity in glycolysis and regulation by acetylation. PGK1 function is regulated by acetylation at K323, which enhances PGK1 enzymatic activity, improves glycolytic metabolism and promotes tumor growth. K323 acetylation is regulated by PCAF and SIRT7, allowing stimulation or inhibition of PGK1 function. P300/CBP-associated factor (PCAF) is a transcriptional coactivator associated with p53 promoting acetylation of PGK1. Conversely, NAD-dependent deacetylase sirtuin 7 (SIRT7), a nuclear enzyme, suppressed DNA recombination. GAPDH: glyceraldehyde 3 phosphate dehydrogenase. PGK1: Phosphoglycerate kinase 1 (Adapted from [76]).

**Figure 6:**
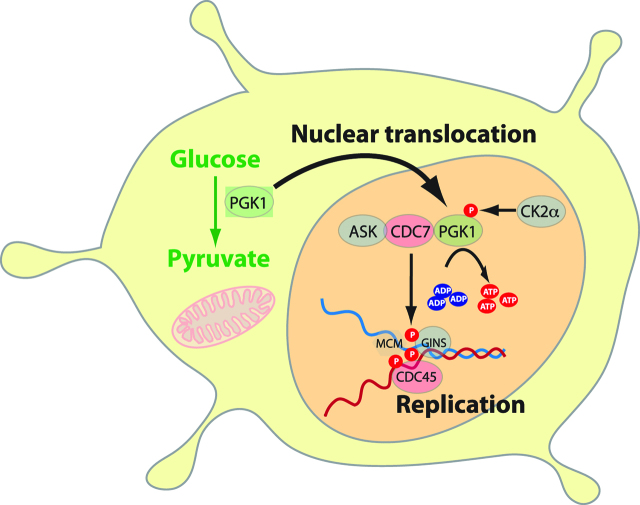
Role of PGK1 as a transcription factor. PGK1 is translocated into the nucleus. PGK1 is phosphorylated by casein kinase 2α (CK2α) at S256 under control of EGF-R and ERK 1/2. Phosphorylated PGK1 binds to Cell Division Cycle 7-related protein kinase (CDC7) and converts ADP to ATP. PGK1 also releases ADP inhibition on CDC7, which promotes DNA replication, cellular proliferation and tumourigenesis (Adapted from [73]).

Apart from their metabolic role in rapid generation of ATP and lactate, the glycolytic enzymes also have nuclear effects promoting EMT and carcinogenesis as transcription factors (reviewed in [32]). For example, aldolase-A, a glycolytic enzyme which catalyses the conversion of fructose-1,6-bisphosphate to glyceraldehyde 3-phosphate and DHAP, also upregulates N-cadherin and vimentin and downregulates E-cadherin [48]. Another example is alpha-enolase (ENO1): ENO1 is upregulated by HIF under hypoxic conditions and has been shown to promote cell proliferation, cycle progression, cancer migration and invasion [71]. PGK1 also ‘moonlights’ as a nuclear promoter of DNA replication. Nuclear PGK1 is phosphorylated by EGFR- and ERK-activated casein kinase 2α (CK2α). The phosphorylated PGK1 interacts with kinase cell division cycle 7 (CDC7) by converting ADP to ATP, thus removing the inhibitory effect of ADP on CDC7-S-phase kinase (ASK) activity. This enables DNA replication, cell proliferation and tumourogenesis [72] (Figure 5,6).

## Phosphoglycerate kinase 1 (PGK1) in cancer proliferation

Glycolytic enzymes, in particular pyruvate kinase (PK) and PGK 1 (PGK1) play central roles in cancer cell proliferation. PK catalyses the reaction of dephosphorylation of phosphoenolpyruvate to pyruvate and generates one adenosine triphosphate (ATP) molecule at the same time [73]. PGK1 is an enzyme of the glycolytic pathway, which is regulated by hypoxia-inducible factor-1α (HIF-1α). Its role has been described in tumour progression and metastasis in several malignancies. PGK1 transfers a phosphate group from 1,3-biphosphoglycerate to ADP, which forms ATP and 3-phosphoglycerate. PK and PGK1 are the only enzymes in the glycolytic pathway which generate ATP, hence their importance for continued cellular energy production under hypoxic conditions [74] (Figure 5).

Acetylation of PGK1 promotes liver cancer cell proliferation and tumourigenesis [48]. Increased expression of PGK1 in colon cancer tissue is associated with metastasis. Several genes induced by PGK1 could account for cell migration, mainly early growth response (EGR1) and Cysteine Rich Angiogenic Inducer 61 (CYR61) together with the transcription factors FOS and JUN [76]. Overexpression of PGK1 is known to increase the expression of CXCR4. CXCR4 on its part can increase CXCL12 expression. Elevated levels of CXCR4 and CXCL12 are associated with an increase in the metastatic rate and play an important role in the metastatic homing of malignant cells [77]. PGK1 expression has been shown to be upregulated in human pancreatic ductal adenocarcinoma, paclitaxel resistant breast cancer, adriamycin-resistant leukemic K562 cells, radioresistant astrocytomas and multidrug-resistant ovarian cancer cells as well as in metastatic hepatocellular, colon and gastric carcinoma cells [74, 78] (Figure 5).

## Overexpression of PGK-1 in peritoneal metastasis

In previous studies, we found PGK1 to be significantly overexpressed in PM of gastric origin. This is in concordance with research showing that hypoxia, EGFR activation, and expression of K-Ras G12V and B-Raf V600E induce mitochondrial translocation of PGK1. This translocation is mediated by ERK-dependent PGK1 S203 phosphorylation and subsequent PIN1-mediated cis–trans isomerization. Mitochondrial PGK1 uses ATP as a phosphate donor to directly phosphorylate and activate pyruvate dehydrogenase kinase isozyme 1 (PDHK1) at Thr338. The subsequent PDHK1-mediated phosphorylation of pyruvate dehydrogenase E1α at Ser293 inhibits the pyruvate dehydrogenase complex (PDC) and the conversion of pyruvate and CoA to acetyl-CoA in the mitochondria. This inhibition reduces mitochondrial pyruvate utilization, suppresses reactive oxygen species production, increases lactate production from pyruvate in the cytosol and promotes tumourigenesis [78]. Conversely, depletion of PGK1 with shRNA in hepatocellular carcinoma (HCC), resulted in a significant decrease in tumour proliferation, reversal of extracellular acidification and impairment of cancer cell glycolysis [78]. Thus, mitochondrial pyruvate metabolism suppressed by PGK1 is coupled with the upregulation of glycolytic gene expression mediated by nuclear PKM2 to promote the Warburg effect and tumourigenesis.

In a further previous study, when profiling gene expression in diffuse primary gastric cancer, we observed a significant overexpression of PGK1, the chemokine CXCR4 and its ligand CXCL12 in PM patients. PGK1 was expressed not only in the cytosol, but also in the nucleus of malignant cells, both in the preclinical model and in human gastric cancer [79]. This finding invalidates overexpression of PGK1 in PM as being just a metabolic epiphenomenon and suggests ‘moonlighting’ functions of PGK1: first, as a protein kinase in mitochondria and second, as a DNA replication factor in the nucleus of metastatic cells (see below: role of PGK-1 as a transcription factor). CXCR4 positivity of primary gastric carcinoma also significantly correlated with the development of PM, suggesting that the CXCR4/CXCL12 axis might play an important role in the development of PM from gastric carcinoma. If this is confirmed, then CXCR4 may be a potential therapeutic target to prevent peritoneal metastatic homing of cancer cells of gastric origin [80].

In another prior study simulating human gastric cancer behaviour by orthotopic tumour implantation using a xenograft model in the nude mice, we evaluated the course of tumour growth by MR-imaging and PET/MRI fusion. Elevated PGK1 expression significantly increased invasive and metastatic behaviour of implanted gastric tumours. MRI/PET-imaging of tumour growth and metastasis in *in-vivo* and subsequent *ex-vivo* models were concordant with immunohistochemical PGK1 staining [81]. Notably, differences in PGK1 mRNA expression between gastric cancers disseminating or not disseminating to the peritoneum were comparable with values in our orthoptic xeno-transplanted *in-vivo* mouse model [81].

## PGK-1 as a nuclear transcription factor

In previous studies, we observed increased nuclear expression of PGK1 both in the orthotopic xenograft mouse model and in clinical biopsies of PM of gastric origin. Whereas cytoplasmic activity of PGK1 is compatible with its canonical activity as a glycolytic enzyme, nuclear translocation of PGK1 suggests a non-canonical activity as a transcription factor in metastatic cells (Figure 6) [82].

## Conclusion and outlook

Taken together, these results suggest that PGK1 is crucially involved in the glycolytic switch, cytosolic glycolysis, lactate production, extracellular acidification, autophagy and resistance to anoikis of tumour cells shed into the peritoneal cavity. Overexpression of PGK1 in PM does not appear to be just an epiphenomenon associated with increased glucose metabolism in cancer. Our previous functional experiments *in-vitro* and in the animal model showed that PGK1 knock-out or inhibition with shRNA is effective in controlling the development and growth of PM of gastric origin, establishing a causal role of PGK1 in this development. PGK1 promotes DNA replication and cellular prolferation once translocated into the cell nucleus. This is confirmed by the nuclear translocation of PGK1 during the metastatic process.

The moonlight functions of glycolytic enzymes in carcinogenesis, and in particular the involvement of PGK1/CXCR4 in gastric peritoneal metastasis might have important therapeutic potential. Inhibition of PGK1 might be synergistic in potentiating the cytotoxic effects of systemic chemotherapy on PM. However, the ubiquitous presence of PGK1 and the elevated risk of systemic toxicity may preclude intravenous delivery. A technique to circumvent this limitation might be to aerosolize shPGK1 into the peritoneal cavity. This approach appears attractive since aerosolization of siRNA conserves transfection potential [83] and locoregional delivery may prevent systemic metabolic side-effects. Targeting non-canonical PGK1 function as a promoter of inflammation and immune response is another future research avenue.
